# Our Broadest Future Is Yet before Us

**Published:** 1897-07

**Authors:** 


### EDITORIAL.

### OUR BROADEST FUTURE IS YET BEFORE US.

Since the advent of the bicycle and electric car the statement has been made that horses will soon become a thing of the past, and veterinary services will be required no longer. The same statement was made when steam roads were first operated, but experience shows that far more horses were required to carry on the commerce that developed as a result of improved facilities for transportation than could be used under the old system, when all inland travel was dependent upon horse-power, and local wants were met almost entirely by local products. While it is true that some horses have been displaced by mechanical agencies, it is also true that the demand for horse-labor in other directions has increased correspondingly and in proportion to the activity of the commerce of the country. For the past few years certain classes of horses have been very cheap, ana this is directly attributable to the sudden increase in their production, an increase that was far beyond all possible demands, and to the general depression of all business which greatly reduced the demand for horse-labor as well as for that of man. But good horses are and always have been in constant demand at remunerative prices, and all well-informed horsemen admit that the demand for horses of good quality is greater than the supply. Horse-breeding was such an exceedingly profitable occupation a few years ago, and there was such an active demand for horses of a very inferior type, that anyone with a little capital could engage in the rearing of horses and make money. That period might justly be compared to the boom-period of a Western town or to the rush into any branch of industry that is apparently simple, easy to carry on, and promises large returns. But now the business of breeding horses is established on a better, sounder, and firmer basis. The breeders of this country are beginning to understand that it is only by the exercise of much knowledge, good judgment, and constant watchfulness that horses of superior quality can be produced, but that when these requisites are properly applied the reward is satisfactory. Horses will always be used for draught-work in cities and in country; they will always be used for riding and driving; and racing, the sport of kings, will always continue.

But the horse might become entirely extinct, and an enormous field for the employment of veterinary knowledge and skill would still remain. According to the statistics of 1896 the horses of the United States number 15,125,057, and are valued at more than $500,000,000, while the mules of the country are worth $103,000,000 more. The cattle of the United States number 48,200,000, and are worth $873,000,000. The sheep are worth over $65,000,000, and are rapidly increasing in number and value. The swine number about 43,000,000, and are worth more than $186,000,000. The total value of the several classes of live-stock mentioned amounts to $1,727,926,084. This total is considerably less than it has been for some years, and than it will be when the general business conditions improve and live-stock values respond to the resulting stimulation. The export trade of the United States bears principally upon the live-stock industry, and exports of horses, cattle, meats, and dairy-products are increasing from year to year.

No, the field of the veterinarian is not bounded by any prospective limitation of the horse's usefulness. It includes the cultivation, care, and protection of animals that supply us with our most nutritious and expensive foods; with many of the luxuries as well as necessaries of our diet; with warm clothing for our body, head, hands, and feet; with many of the pleasures and comforts of life; with agreeable, healthful, and elevating recreation, as well as those that furnish the most common means of transporting freight.

